# Spatial accessibility and equity of emergency medical services: Evidence from Ningbo

**DOI:** 10.1371/journal.pone.0362204

**Published:** 2026-11-04

**Authors:** Minxia Fu, Jieyi Guan, Yixi Zheng, Xingchuan Gao, Wenwan Jin, Pengtao Wang

**Affiliations:** 1 School of Geography and Remote Sensing, Ningbo University, Ningbo, China; 2 School of Tourism, Xi’an International Studies University, Xi’an, China; Zhejiang University, CHINA

## Abstract

**Background:**

Timely access to emergency medical services (EMS) is essential for improving survival outcomes and public health. In rapidly urbanizing Chinese cities, disparities in EMS accessibility are increasingly shaped by the spatial distribution of hospitals, population concentration, transport infrastructure, and built environment conditions. However, relatively few studies have simultaneously compared emergency and non-emergency medical accessibility and examined their spatial equity and influencing factors in medium-sized coastal cities. This study evaluates the spatial accessibility, equity, and clustering characteristics of EMS in Ningbo, China, and identifies the key built environment factors influencing these patterns.

**Methods:**

The shortest travel time model, based on API data, was employed to measure EMS accessibility for both emergency (ambulance) and non-emergency (public transport) scenarios across five core urban districts of Ningbo. The Lorenz curve and Gini coefficient were applied to assess healthcare equity. We used global Moran’s I and local indicators of spatial association (LISA) to explore spatial clustering and a multiscale geographically weighted regression (MGWR) model to analyze spatial heterogeneity in influencing factors across built environment dimensions.

**Results:**

Both emergency and non-emergency medical services exhibited significant spatial disparities, decreasing gradually from the city center toward the periphery. The Gini coefficients for emergency and non-emergency models were 0.5156 and 0.4898, respectively, indicating substantial inequity. Strong spatial clustering was observed (global Moran’s I = 0.846, *p* < 0.01), with high-value clusters concentrated in central districts and low-value clusters in mountainous outskirts. MGWR results revealed that accessibility was positively affected by the number of bus stops, road density, bus route density, and distance to the Central Business District (CBD), but negatively impacted by building density and development intensity. The MGWR model’s explanatory power (R^2^ = 0.584) demonstrated significant spatial heterogeneity in these relationships. Building density and bus route density exerted the strongest local effects among the eleven built environment indicators.

**Conclusion:**

EMS accessibility in Ningbo exhibits evident spatial inequality and clustering characteristics. Peripheral and mountainous areas remain disadvantaged, especially under non-emergency public transport conditions. Improving road connectivity, expanding public transit coverage, optimizing hospital and EMS resource allocation, and strengthening services in underserved areas can help reduce healthcare accessibility inequities. These findings provide an empirical foundation for optimizing EMS resource allocation for urban policymakers as well as offer practical insights for other rapidly urbanizing cities facing similar challenges.

## Introduction

With the rapid acceleration of global urbanization, emergency medical services (EMS) have become fundamental for safeguarding public health and safety. Enhancing EMS efficiency is directly aligned with the United Nations’ 2030 Sustainable Development Goals (SDGs), particularly Good Health and Well-being (SDG 3) and Sustainable Cities and Communities (SDG 11). In the context of China’s high urbanization and aging population, EMS resources have emerged as critical indicators of social welfare and urban livability. This is especially true in densely populated urban environments [1], where the extent of EMS coverage dictates the ability to secure the “golden rescue time,” which profoundly impacts public survival outcomes and the overall quality of urban life.

EMS is a critical component of urban public health systems and plays an indispensable role in emergency response, spatial resource allocation, and ensuring timely access to care [2,3]. Accessibility was originally proposed by Hansen [4] as a key indicator of the coverage and convenience of public services, reflecting the ease or difficulty of reaching a specified location. Among the various performance metrics, transport time—the travel duration from the incident site to the hospital—stands out as a core indicator of EMS efficiency. Transport time reflects urban traffic conditions and EMS coverage more objectively than response time or on-scene treatment time. Because it directly determines whether rescue teams can arrive at a scene promptly and deliver effective care, its impact on EMS efficiency surpasses that of traditional factors such as ambulance availability. Equity in EMS accessibility is a vital dimension in evaluating public health system effectiveness, as it directly affects the health rights of vulnerable populations.

Previous studies have shown that unequal spatial allocation of medical resources can exacerbate social vulnerability and health risk exposure, especially among suburban and low-income populations [5–7]. Empirical research in China and other countries has demonstrated that medical accessibility often presents a core-periphery gradient and is closely related to transport infrastructure and population distribution [6,7]. Therefore, evaluating EMS systems requires not only measuring travel time efficiency but also examining whether accessibility is equitably distributed across population groups. Research on EMS accessibility has thus increasingly become a pivotal topic for optimizing public service resource allocation and enhancing emergency response efficiency [8].

Traditional accessibility measurement models include Euclidean distance, spatial impedance models, gravity models, opportunity models, and utility models, and more recent multimodal accessibility measures [4,9–11]. The two-step floating catchment area (2SFCA) method has commonly been used to assess EMS accessibility [12–16], as it emphasizes the spatial distribution of and competition for medical resources. Time-based accessibility—how quickly patients can obtain medical services—is more critical than distance-based measures with respect to passive healthcare seeking, such as EMS.

EMS accessibility is influenced by a wide range of factors. First, the structural layout of the transportation network—including its topological connectivity and hierarchical integrity, as well as the road design of the urban transport system—directly affects route selection for emergency response, thereby impacting EMS efficiency [17–19]. Second, dynamic traffic conditions place critical constraints on accessibility assessments. Real-time traffic flow, such as congestion [20,21], time-varying transport conditions [11], or the impedance of severe weather events [22], can significantly diminish EMS accessibility. Third, the spatial distribution of healthcare resources plays a decisive role. The quantity, spatial distribution, and service capacity of healthcare resources typically exhibit a gradual decline from central urban areas outward, resulting in corresponding changes in healthcare accessibility [6,23–25]. Finally, socioeconomic factors such as the urbanization rate, per capita GDP, and population density also influence EMS accessibility outcomes [26,27]. While not directly determining physical efficiency, it does have an indirect impact on it.

Research on EMS accessibility still faces certain limitations. First, many studies focus primarily on one-way travel time [28,29], often overlooking the entire duration of EMS operations. Second, research within China tends to concentrate on megacities such as Beijing [22,30], Shanghai [21,23], and Wuhan [31], whose dense medical networks and transport systems differ substantially from medium-sized cities, limiting the transferability of their conclusions. Third, the majority of studies emphasize the spatial distribution of EMS accessibility [12], with comparatively few conducting an in-depth exploration of the underlying influencing factors.

Ningbo City has a registered elderly population of nearly 2 million, accounting for approximately 30% of the total population in 2025. Ningbo City is the economic center of the southern Yangtze River Delta and a modern international port city. It is estimated that by 2050, the global population aged 60 and above will be almost double its current size. This striking change in population structure will bring about significant challenges across all sectors of society, with healthcare service systems hit particularly hard [32]. Although the number of emergency medical stations in Ningbo has increased to 159, the distribution of emergency resources remains uneven. More than half of the townships in districts such as Haishu still lack emergency stations, severely impacting EMS accessibility.

In summary, despite the substantial progress that has been made in the study of EMS accessibility, some research gaps remain. This study focused on the accessibility of both emergency and non-emergency medical services, examining equity in healthcare access based on the resident population. We analyzed the observed clustering phenomena and heterogeneity. Findings reveal significant spatial disparities in accessibility to both emergency and non-emergency medical services, with a clear diminishing trend from the city center to suburban areas. Significant inequalities exist in healthcare equity. Spatial clustering and heterogeneity are pronounced, with factors such as proximity to bus stops and distance from the CBD showing positive influences. This research aims to provide an evidence-based foundation for improving EMS accessibility and optimizing the allocation of medical resources in Ningbo, while also offering valuable insights for other cities and regions facing similar challenges.

## Research area, data sources and research methods

### Research area

This study focused on Ningbo, located in the northeastern part of Zhejiang Province at the southern edge of the Yangtze River Delta. As of the end of 2023, Ningbo administers ten administrative divisions; due to data availability, five central urban districts were selected as the study area, covering approximately 25.5% of the total area (Fig 1). The Implementation Plan for Promoting the Modernization of Health and Medical Services in Ningbo (2023–2027) aims to establish a more comprehensive and efficient EMS system by optimizing medical resource allocation and improving healthcare service quality to enhance public health outcomes [33].

**Fig 1:**
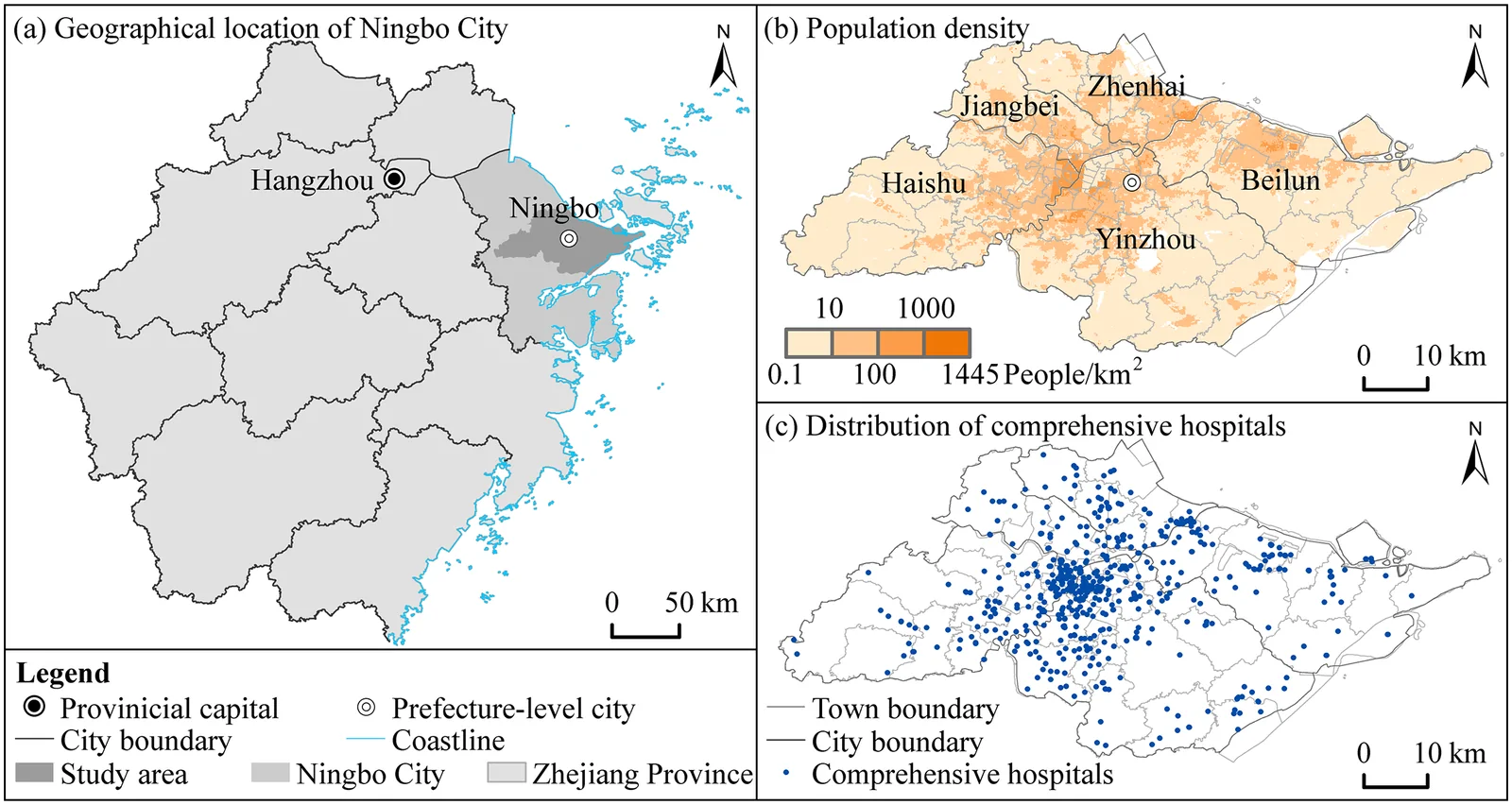
Population and hospital distribution in downtown Ningbo. Hospitals: Ningbo Municipal Government Open Data Platform; population: WorldPop (CC BY 4.0); administrative boundaries: Natural Earth (public domain); basemap: ©OpenStreetMap contributors (ODbL).

As an economically developed city located in the southern Yangtze River Delta, Ningbo has a population density of 633.83 persons of km^2^ and an urbanization rate of 79.9% (2023). However, its EMS system still faces significant challenges. First, a structural imbalance exists between the supply and demand of medical resources. The city has only ten tertiary A hospitals, and the number of health technicians per 1,000 residents is 9.55, which is considerably lower than in mega-cities such as Beijing (14.31) and Shanghai (9.88) [34]. Second, the spatial distribution of medical resources is uneven; for example, tertiary A hospitals are primarily concentrated in central districts (Yinzhou). Consequently, addressing these disparities in both spatial and temporal dimensions is necessary for Ningbo to improve public health and overall well-being [23,35,36].

### Data sources

This study utilized the following primary data sources:

(1) Healthcare Facility Data: Information from medical institutions was obtained from the 2023 update on the Ningbo Municipal Government Open Data Platform (http://data.ningbo.gov.cn). Village clinics, cosmetic outpatient centers, and dental specialty clinics were filtered out, leaving a total of 37 general hospitals for analysis.
(2) Road Network Data: Road network data were sourced from the Resource and Environmental Science Data Center (www.resdc.cn) and the OpenStreetMap (OSM) dataset (https://www.openstreetmap.org). OSM offers more detailed and comprehensive road information than other sources.
(3) Population Data: Population raster data with a 100-meter resolution for the year 2020 were obtained from the WorldPop website (https://www.worldpop.org). This dataset provides a more accurate representation of the spatial distribution of the population than administrative statistical data.
(4) Administrative Boundary Vector Data: Vector data for administrative boundaries were sourced from Natural Earth (https://www.naturalearthdata.com). The city- and district-level attributes of the administrative divisions were extracted from the 1:10m cultural vectors and refined for use in this study.
(5) Shortest travel time to medical care: Travel time metrics for 9,029 regular grids covering central Ningbo were computed exclusively via open-source routing algorithms based on Natural Earth road network layers. We divided the central urban area of Ningbo into 9,029 grids and calculated the shortest travel time from each grid to a general hospital for both private cars and public transportation.

### Methods

#### Healthcare accessibility.

We categorized healthcare accessibility into two types: emergency medical accessibility (passive healthcare access)—the total travel time for an ambulance’s round trip from a general hospital to an emergency site and back again—and non-emergency medical accessibility (active healthcare access)—the time it takes a resident to travel to a hospital from their home under normal circumstances [11,23,35]. The waiting time and on-site treatment time depend on operational and medical processes and are therefore not included in this study, which focuses exclusively on transportation-based accessibility [23, 31]. It should be noted, however, that actual access to care is influenced not only by travel time but also by institutional capacity, service demand, and case complexity [24]. In peripheral or suburban areas, longer travel distances are often accompanied by longer waiting times due to limited medical resources and fewer specialized facilities [24,25]. Moreover, patients with complex or rare conditions may need to seek care at higher-level or specialized hospitals located in central urban areas [10,31], which further increases both travel and waiting burdens for residents in edge regions. These factors may amplify spatial disparities in real healthcare access beyond what is captured by transportation time alone.

Healthcare travel time was calculated by constructing a 100 m*100 m grid over the central urban area. After preprocessing the five selected districts in Ningbo and removing mountainous regions and water bodies, a total of 9029 grid cells were obtained (Fig 2). The centroid of each grid cell was used as the destination point, while general hospitals served as the origin points. Ambulance transport was used as the mode of travel in emergency medical scenarios, whereas public transportation was the assumed mode of travel in non-emergency scenarios. Ambulance transport was used as the representative travel mode for emergency medical scenarios because emergency patients are generally transported through pre-hospital emergency medical systems, in which road-based motorized travel is the principal means of connecting emergency sites with hospitals. In this study, driving travel time was therefore used as a proxy for the road-network component of ambulance transport. For non-emergency medical scenarios, public transportation was selected to represent routine healthcare travel because such trips are planned rather than dispatched through the EMS system and residents may rely on ordinary urban transport services to reach hospitals. More importantly, the two modes were not intended to represent all possible travel behaviors. Instead, they were used to construct two contrasting transportation scenarios corresponding to emergency and routine healthcare access. The resulting estimates should therefore be interpreted as scenario-based transportation accessibility rather than the actual travel behavior of every resident. Other travel modes, such as bicycles or electric scooters, were excluded from consideration. The shortest travel time from hospitals to each grid centroid was calculated for both scenarios using the application programming interface (API) [35,36]. For emergency medical travel, the total time required for a round trip (from hospital to incident site and back) was considered, because EMS accessibility is strongly affected by both transport time and the spatial distribution of emergency resources [31,37]. The resulting values reflect the transportation accessibility for both emergency and non-emergency medical services (Fig 3). For each grid cell, the minimum travel time to a general hospital was used to characterize transportation-based healthcare accessibility under the corresponding travel scenario, thereby reflecting the relative ease of reaching hospital services across space. The calculation formula is as follows:


$$\begin{array}{c} A_{ij}^{e}=\text{2}*Min(M_{ij}T_{ij}^{e}) \end{array}$$ (1)

$$\begin{array}{c} A_{ij}^{n}=Min\left(M_{ij}T_{IJ}^{n}\right) \end{array}$$ (2)

**Fig 2:**
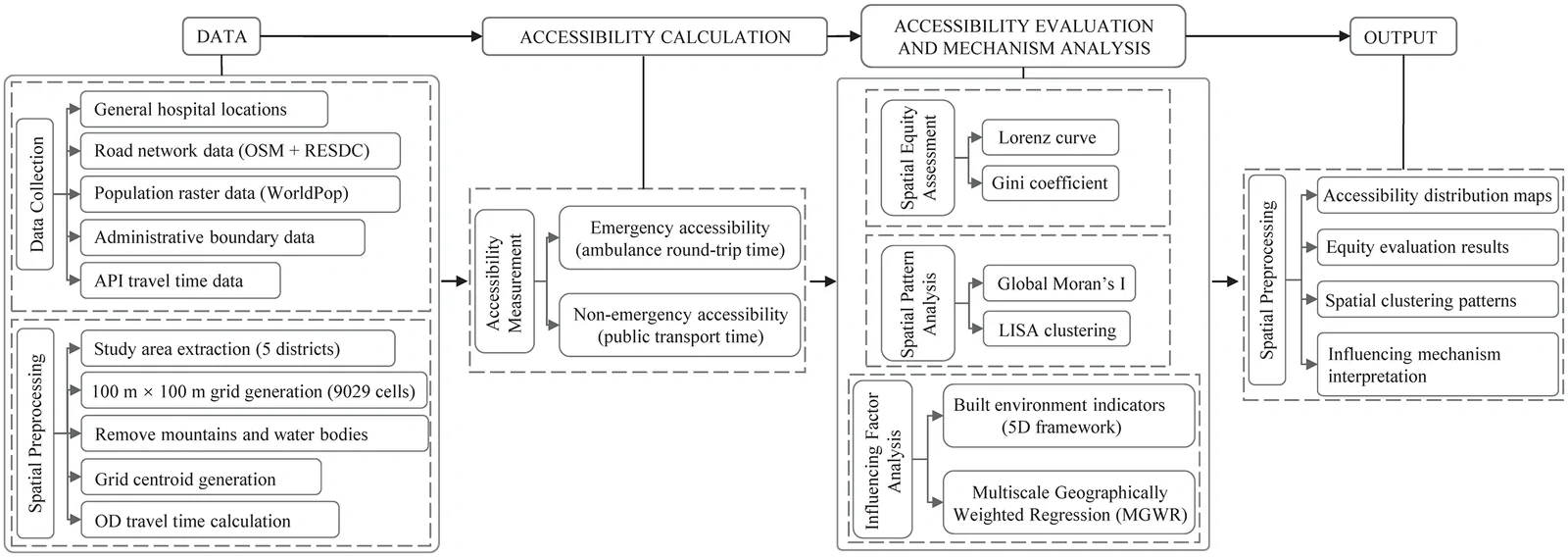
Methodological flowchart.

**Fig 3:**
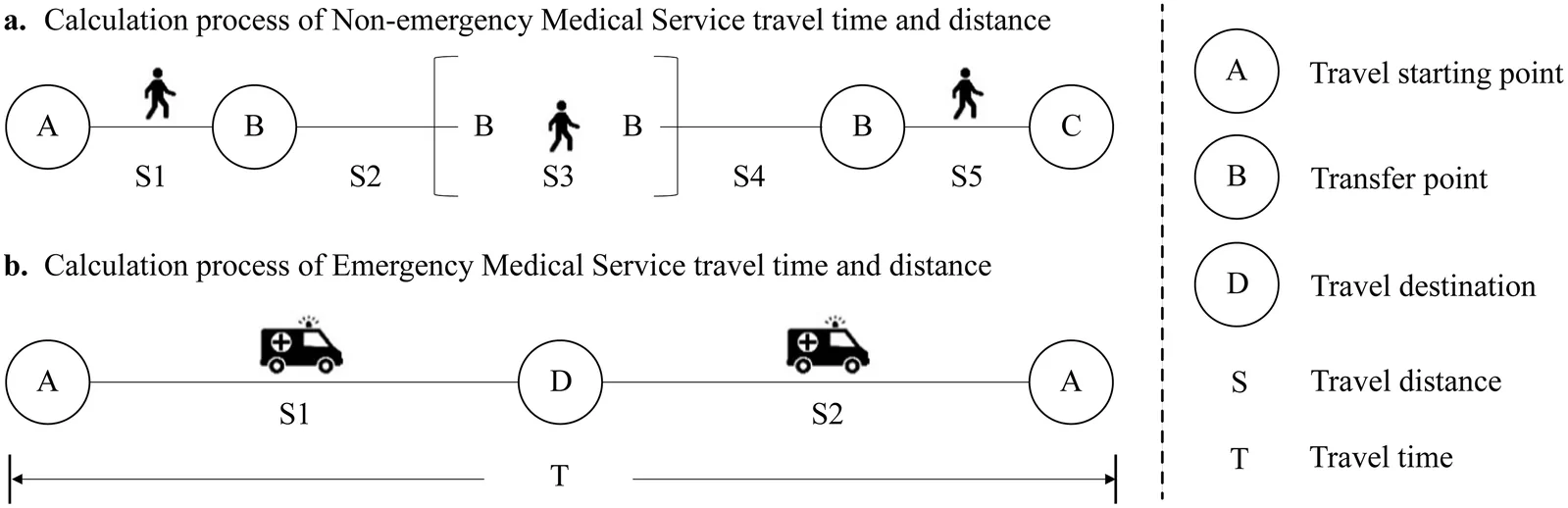
Medical accessibility calculation.

where *T*_*ij*_^*e*^ and *T*_*ij*_^*n*^ represent the travel time from grid cell *i* to medical facility *j* in the emergency and non-emergency medical scenarios, respectively. *M*_*ij*_ denotes the weight of interaction between grid cell *i* and medical facility *j*, which is set to 1 in this study. *A*_*ij*_^*e*^ and *A*_*ij*_^*n*^ represent the time-based accessibility of medical facility *j* to grid cell *i* under emergency and non-emergency medical scenarios, respectively.

#### Lorenz curve and Gini coefficient.

The Lorenz curve and its corresponding Gini coefficient are widely used indicators for measuring equity [12,16]. The Lorenz curve is used in economics to illustrate the degree of equality or inequality in income distribution within a country, while the Gini coefficient is one of the most frequently used measures of income disparity. These tools have also been extensively applied in transport equity research [38, 39]. We employed both the Lorenz curve and Gini coefficient in this study to assess the spatial equity of emergency medical accessibility in Ningbo. First, the population size of each grid cell was processed. Then, based on the level of medical accessibility in each cell, the values were matched with corresponding population sizes to establish a functional relationship. This allowed for the construction of a Lorenz curve illustrating the correspondence between the cumulative population distribution and the cumulative distribution of the accessibility indicator. In this study, population is treated as a spatial distribution variable rather than a socio-demographic classification [40]. Differences among social groups (e.g., poverty status or vulnerable populations) are not distinguished; therefore, the resulting Lorenz curve reflects spatial equity in accessibility rather than demographic or social equity. The Gini coefficient ranges from 0 to 1, with values closer to 0 indicating higher equity and values closer to 1 indicating lower equity. The approximate formula for calculating the Gini coefficient is as follows:


$$\begin{array}{c} G=\text{1}-\sum_{i=\text{1}}^{k}\left[\left(x_{i}-x_{i-\text{1}}\right)\times \left(y_{i}-y_{i-\text{1}}\right)\right] \end{array}$$ (3)

where *G* denotes the Gini coefficient, *x*_*i*_ represents the cumulative proportion of the population of unit *i* relative to the total population (with *x*_*0*_ = 0, *x*_*1*_ = 1), *y*_*i*_ represents the cumulative proportion of the accessibility in unit *i* relative to the total accessibility (with *y*_*0*_ = 0, *y*_*1*_ = 1), and *k* refers to the total number of spatial units. In the literature, the Gini coefficient is commonly interpreted as follows: if *G* ≤ 0.2, it indicates an extremely low index level (highly average), *G*∈[0.2, 0.29) indicates a low index level (relatively average), *G*∈[0.3, 0.39) indicates a moderate index level (relatively reasonable), *G*∈[0.4–0.59) indicates a high index level (significant difference), and *G*∈[0.6, 1] indicates an extremely high index level (significant difference).

#### Spatial autocorrelation.

In accordance with the First Law of Geography [41], we employed spatial autocorrelation analysis to evaluate spatial imbalances in medical accessibility [23,27,36]. Specifically, we used the global Moran’s I index to identify overall spatial correlation and clustering patterns and the local indicators of spatial association (LISA) to detect localized clustering relationships and spatial distribution patterns [42]. We used the average shortest travel time within each township to represent the shortest travel time for that township or subdistrict, which served as the basis for the spatial autocorrelation analysis. Moran’s I ranges from −1–1: values >0 indicate positive spatial correlation (stronger with larger values), values <0 indicate negative correlation (greater spatial disparity with smaller values), and 0 indicates no spatial correlation. The global Moran’s I index is calculated using the following formula:


$$\begin{array}{c} \text{I}=\frac{\text{N}}{{\text{S}}_{\text{0}}}\times \frac{\sum_{\text{i}=\text{1}}^{\text{N}}\sum_{\text{j}=\text{1}}^{\text{N}}{\text{w}}_{\text{ij}}({\text{X}}_{\text{i}}-\text{X})({\text{X}}_{\text{j}}-\text{X})}{\sum_{\text{j}=\text{1}}^{\text{N}}{\left({\text{X}}_{\text{i}}-\text{X}\right)}^{\text{2}}} \end{array}$$ (4)

where *N* is the number of townships, *X*_*i*_ is the value of research unit *i*, *X*_*j*_ is the value of research unit *j*, *i* ≠ *j*, *w*_*ij*_ represents the spatial relationship between *i* and j, and if *i* is adjacent to *j*, *w*_*ij*_ is equal to 1. If *i* is not adjacent to *j*, then *w*_*ij*_ is equal to 0.

The LISA for each township’s value reflects the spatial aggregation of that township’s value that is close to its spatial proximity, and the global spatial correlation coefficient can be decomposed into spatial autocorrelation over each region. The LISA analysis reveals local spatial heterogeneity, including four clustering types: high-high, high-low, low-low, and low-high. High-high and low-low clusters indicate small differences between a region and its surrounding areas, reflecting a spatial concentration of relatively high or low values. In contrast, low-high and high-low clusters suggest discrepancies between a region and its neighbors. Furthermore, the local spatial autocorrelation results can be clearly illustrated using visualization charts. For the *i*-th study unit, Moran’s I LISA formula is:


$$\begin{array}{c} I_{i}=\frac{\left(x_{i}-x\right)}{\sum_{i}{\left(x_{i}-x\right)}^{\text{2}}/n}\sum_{j}W_{ij}(x_{i}-x) \end{array}$$ (5)

#### Multiscale geographically weighted regression analysis.

The multiscale geographically weighted regression model (MGWR) is a statistical modeling approach used to analyze spatial non-stationarity, which is particularly suitable for examining spatial heterogeneity in the relationships between dependent and explanatory variables [43, 44]. Compared to the traditional geographically weighted regression (GWR) model, MGWR introduces the concept of multiple spatial scales, allowing each explanatory variable to operate under its own bandwidth and level of spatial smoothing [43,44]. This enables a more refined depiction of spatial heterogeneity characteristics [43]. By applying locally weighted least squares regression, MGWR reveals how the influence of explanatory variables on the dependent variable varies across geographic locations. The general regression equation is expressed as:


$$\begin{array}{c} y_{i}=\beta_{\text{0}}({\text{u}}_{i},{\text{v}}_{i})+\sum_{k=\text{1}}^{p}\beta_{k}(u_{i},v_{i})x_{ki}+\varepsilon_{i} \end{array}$$ (6)

where *(u*_*i*_*, v*_*i*_*)* is the geographic coordinate, *y*_*i*_ is the dependent variable, *x*_*ki*_ is the explanatory variable, *β*_*k*_*(u*_*i*_*, v*_*i*_*)* is the local regression coefficient of the explanatory variable *x*_*k*_ at *(u*_*i*_*, v*_*i*_*)*, and *ε*_*i*_ is the residual.

The estimation of the regression coefficients *β*_*k*_ for different explanatory variables *x*_*k*_ at each point uses different bandwidths *b*_*k*_, that is:


$$\begin{array}{c} W_{k}(u_{i},v_{i})=exp\left(-\frac{d_{ij}^{\text{2}}}{b_{k}^{\text{2}}}\right) \end{array}$$ (7)

where *W*_*k*_ is the weighting matrix for the explanatory variable *x*_*i*_ at location *i*, and *d*_*ij*_ represents the geographic distance between locations *i* and *j*.

### Construction of the influencing factors indicator system

The “3D” framework (Density, Diversity, and Design), proposed by Cervero and Kockelman [45], emphasizes the impact of multidimensional built environment characteristics. This framework has been widely used in early research and serves as a foundational model for analyzing the relationship between the built environment and travel behavior. However, scholars have increasingly recognized the limitations of this framework in explaining complex urban environments. Ewing and Cervero [46] expanded this model, proposing a more comprehensive “5D” built environment framework. The “5D” built environment indicator system has become a classical framework for measuring and analyzing the characteristics of urban form [47]. It highlights the influence of urban morphology on factors such as residents’ travel behavior, health, and accessibility. In addition to the original “3D” elements, the expanded framework includes Destination Accessibility and Distance to Transit. In assessing the built environment, the “5D” framework offers clear advantages in terms of dimensional comprehensiveness and systematic indicators [48,49]. We therefore constructed a “5D” indicator system for the built environment in urban Ningbo, aiming to reveal the characteristics and differences of emergency and non-emergency medical services across different dimensions (Table 1) [44–49].

**Table 1:** Establishment of the environmental “5D” indicator system.

5D	Indicator
**Design**	Number of bus stations
	Density of bus routes
	Density of roads
**Diversity**	Degree of land mixing
**Distance**	Distance to CBD
	Distance to bus stops
**Destination Accessibility**	Distance to hospital
**Density**	Intensity of development
	Residential density
	Building density
	Land use efficiency

## Results

### Accessibility of emergency and non-EMS

Based on the concept of the 15-minute living circle, we categorized the accessibility of emergency and non-emergency medical services in urban Ningbo (Table 2), measuring how easily residents in the city center can reach general hospitals via public transportation and ambulance services. Overall, both emergency and non-emergency medical accessibility times increase with distance from the city center (Fig 4), reflecting the concentration of healthcare facilities in urban cores. Despite lower population densities, peripheral townships may face greater vulnerability due to aging populations and limited medical resources [6,7].

**Table 2:** Proportion of emergency medical and non-emergency medical accessible areas with different service radii.

Different modes	<15 min	15-30 min	30-45 min	45-60 min	>60 min
**Non-emergency medical**	1.78%	4.03%	10.40%	15.33%	68.45%
**Emergency medical**	14.68%	42.94%	25.20%	11.99%	5.19%

**Fig 4:**
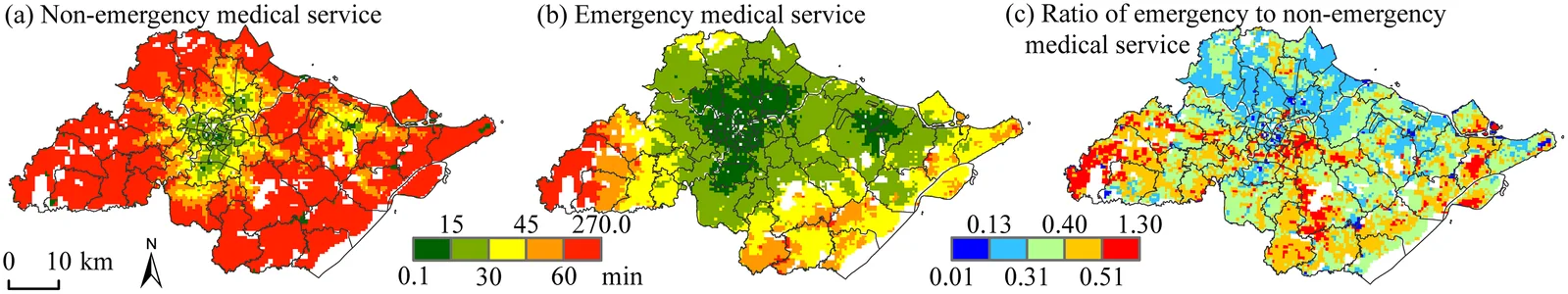
Accessibility of medical care in downtown Ningbo. Shortest travel times computed by the authors via open-source routing on OpenStreetMap road data (ODbL); administrative boundaries: Natural Earth (public domain); basemap: ^©^OpenStreetMap contributors (ODbL).

For non-emergency medical travel, represented by public transportation, only 1.78% of the area is covered by the 15-minute service radius of general hospitals; the coverage of the 30-minute service radius increases to 5.81%, and 68.45% of the area has a medical accessibility time of over 60 minutes. The areas with the best medical accessibility (Fig 4) are concentrated near Gulou, Laowaitan, the Textile City, and Yinzhou Park (≤15 min), followed by the junction of Yinzhou, Haishu, and Jiangbei (15–45 min). Most areas other than the center of the study area exhibit poor medical accessibility (>60min). Bus routes have a significant beneficial effect on medical accessibility [10,11]. For example, the distribution of medical accessibility is better near bus routes 373 and 380 in the southeast of Zhenhai. In terms of emergency medical travel methods (Table 2), the 15-minute service radius of general hospitals covered 14.68% of the area, the 30-minute service radius expanded to 67.62%, and only 5.19% of the area had a medical accessibility time of more than 60 minutes. In terms of spatial layout, the 15-minute service radius covers the built-up area of Ningbo, the 30-minute service radius covers the vast majority of the central urban area, and the areas exceeding 60 minutes are distributed in the western, southern, and eastern mountainous regions. According to the World Health Organization Guidelines for Emergency Medical Services, the target time is 15 minutes in emergencies and 30 minutes in non-emergency situations [50]. The accessible time for emergency medical care should be significantly shorter than that for non-emergency medical care. Our analysis of the differences in the ratio of emergency to non-emergency medical service accessibility times (R-ENESA) in the study area revealed that 89.62% of the area had a relatively reasonable R-ENESA (≤0.5), and 0.10% of the area had a relatively high R-ENESA (> 0.8). These areas may have insufficient coverage of the emergency network or inefficient non-emergency services [3]. The areas with better emergency medical accessibility were concentrated in Jiangbei and Zhenhai.

The following conclusions can be drawn from our analysis of both non-emergency and emergency medical accessibility: medical travel time exhibits a clear spatial differentiation pattern, increasing progressively from the city center toward peripheral areas [7,23]. The central urban area is the historic core of the city, which was developed earlier and hosts a higher concentration of general hospitals, resulting in significantly better medical accessibility compared to outlying regions. Public transportation routes play a notable role in enhancing access to non-emergency medical services, with areas closer to bus lines demonstrating higher levels of accessibility. Compared to non-emergency services, EMS generally features shorter travel times and broader coverage within shorter service radii, and involves a higher number of general hospitals.

### Equity of healthcare based on resident population

According to the 2020 Seventh National Population Census of China, released by the Office of the Leading Group of the State Council for the Seventh National Population Census (2021), the permanent resident population of Ningbo shows a distinct central agglomeration feature [51]. As the area with the highest population density in the city, the central urban area accommodates a large proportion of permanent residents, while the surrounding areas have a relatively sparse population distribution. This unbalanced spatial distribution of the population provides an important basis for the subsequent analysis of medical equity. As can be seen from the Lorenz curve in Fig 5 the accessibility of medical care in the central urban area of Ningbo is relatively unbalanced under both emergency and non-emergency medical modes. Specifically, the Gini coefficient is 0.5156 under the emergency medical scenario and 0.4898 under the non-emergency medical scenario, indicating a slightly greater degree of spatial inequality in accessibility under the emergency scenario. Both values exceed 0.4, suggesting a relatively high level of spatial inequality in healthcare accessibility, although the difference between the two scenarios is modest. This is primarily because most of the medical institutions that can provide EMS are concentrated in the central urban area, and access to medical care is restricted to varying degrees for areas far from the city center. The mismatch between the distribution of medical resources and the spatial distribution of the population leads to significant differences in equity among regions [6,23]. The problem of medical equity becomes more prominent, especially in the emergency medical model, due to the mismatch between the allocation of ambulance resources and the population distribution.

**Fig 5:**
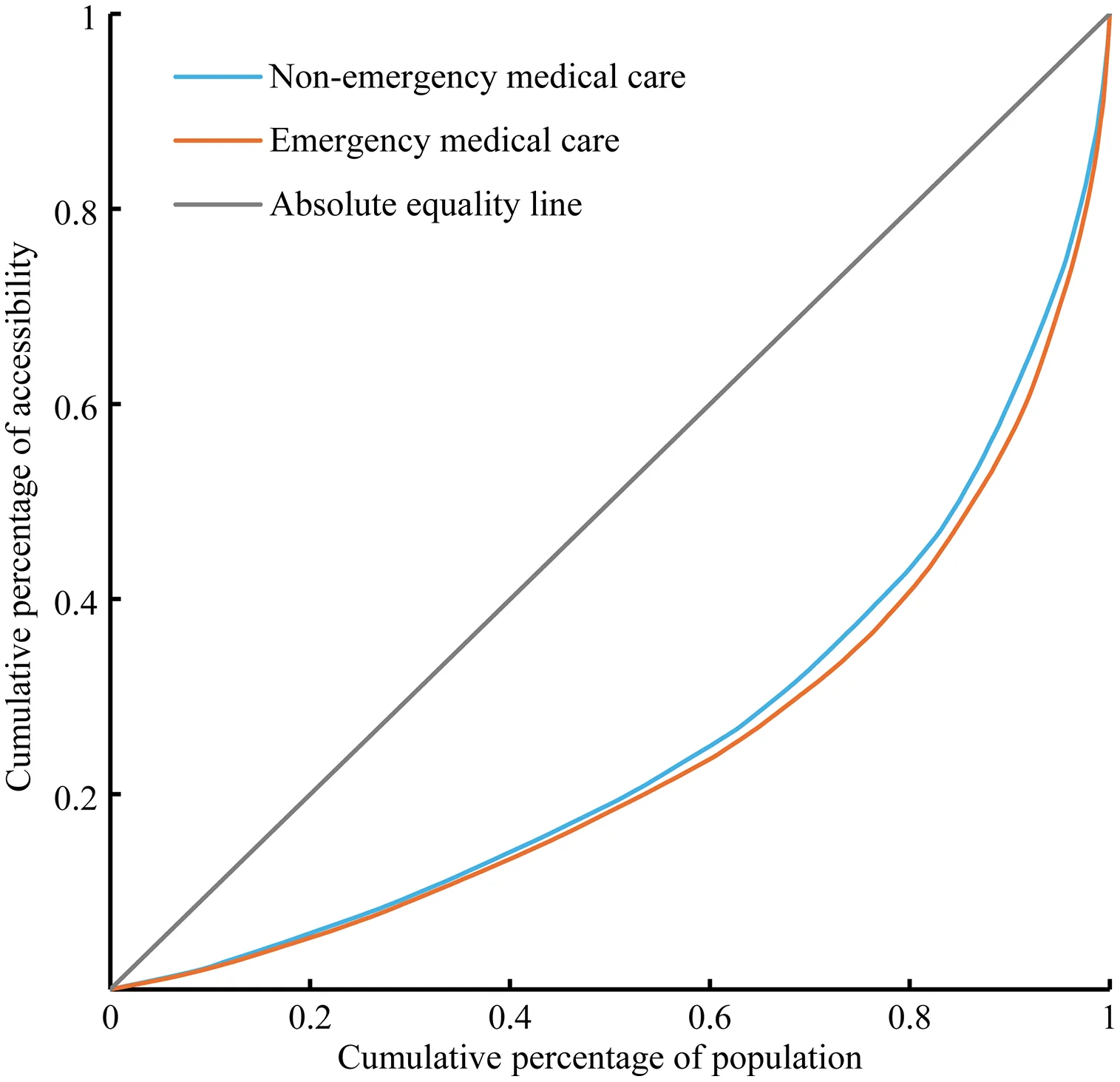
Lorenz curve of medical care accessibility.

A global spatial autocorrelation analysis of accessibility within the study area was conducted to further explore the spatial differentiation characteristics of healthcare accessibility (Fig 6). The results indicate that Moran’s I index for the R-ENESA is 0.846 (*p* = 0.001), suggesting a significant positive spatial correlation. That is, the shortest travel time demonstrates a clear spatial clustering pattern at the township level, indicating that healthcare accessibility exhibits strong spatial aggregation characteristics.

**Fig 6:**
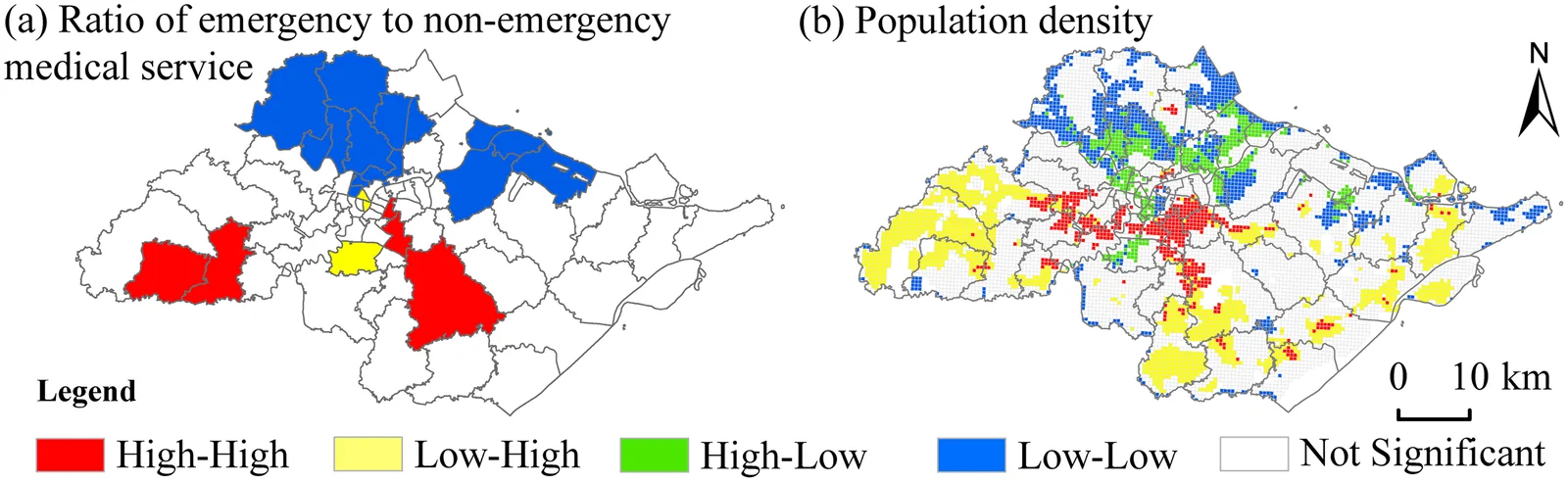
Local spatial autocorrelation of the shortest travel time. Spatial statistics computed by the authors; administrative boundaries: Natural Earth (public domain); basemap: ©OpenStreetMap contributors (ODbL).

In addition, we used LISA to analyze the direct similarity and correlation of medical accessibility in each township and its adjacent townships. The analysis revealed prominent high- and low-value regional clusters in accessibility across the study area with few outliers. The high-high cluster represents a group of areas exhibiting low levels of healthcare accessibility, where both the townships and their surrounding areas are characterized by long travel times to healthcare facilities and minimal differences between emergency and non-emergency medical accessibility. This pattern aligns with complex terrain conditions and underdeveloped economic infrastructure, primarily observed in the western and south-central regions of Ningbo, where hilly landscapes, sparse populations, long distances to general hospitals, and inconvenient transportation networks prevail. The low-low cluster denotes areas exhibiting relatively good spatial accessibility to healthcare services, characterized by short travel times and minimal variability in access across neighboring townships. These regions are distributed across Jiangbei, Zhenhai, and the western part of Beilun, where relatively low population density and relatively high road network density contribute to more efficient access to general hospitals. The low-high cluster appears in only three townships of the economically developed Haishu and Yinzhou, where healthcare accessibility is significantly lower than that of its adjacent areas. Despite its location within an economically developed area, the long travel time to general hospitals and a notable disparity in spatial accessibility indicate a disconnect between the area’s healthcare service provision and its development level. The emergence of this clustering pattern suggests that inadequate public health investment may be a contributing factor at the township level.

The local bivariate Moran’s I index is −0.020 (*p* ≤ 0.05), indicating a spatial negative autocorrelation between shortest travel time and population density. The matching results show that the high-low cluster with better matching is concentrated in the central and southern parts of Zhenhai and Jiangbei. In these areas, the difference in emergency and non-emergency medical accessibility is small, population density is low, and the supply and demand for medical services are relatively balanced. The low-high cluster, where accessibility does not match population distribution, is mainly distributed across the western and southern hilly areas. The high-high cluster is primarily found at the intersection of the economically developed areas of Haishu and Yinzhou, while the low-low cluster is concentrated in the central and northern parts of Jiangbei and Zhenhai.

### Analysis of influencing factors and spatial heterogeneity

This study utilized the MGWR model to investigate the relationship between the built environment and spatial variations in accessibility to general hospitals under emergency and non-emergency medical scenarios. Eleven indicators passed the collinearity test (*VIF* < 10). Except for land use efficiency, land mix degree, and distance to the nearest bus stop, the other eight built environment elements showed strong spatial non-stationarity, with a model goodness of fit R2 of 0.584, indicating that the built environment elements as a whole have greater explanatory power for healthcare equity.

Table 3 summarizes the overall effects of each factor on the R-ENESA for general hospitals. The number of bus stops, distance to the CBD, distance to bus stops, distance to hospitals, bus route density, residential density, and road density have positive effects on the R-ENESA, while building density, development intensity, land use efficiency, and land use mix have negative effects. These findings are consistent with previous studies emphasizing the role of transport infrastructure and travel mode conditions in shaping spatial access to healthcare services [10,11]. The standard deviation of regression coefficients reflects the degree of spatial variation between the R-ENESA and the explanatory variables. The results indicate that land use mix, distance to CBD, and number of bus stops exhibit greater variability, whereas the other variables show relatively lower levels of spatial heterogeneity. Similar spatial heterogeneity in healthcare accessibility has also been observed in large metropolitan settings using GIS-based accessibility analysis [19]. In terms of the absolute values of the mean and median regression coefficients, the four factors with the most influence on accessibility to general hospitals in both emergency and non-emergency scenarios are building density, bus route density, road density, and residential density. This suggests that the impact of built environment characteristics on medical accessibility varies significantly between emergency and non-emergency contexts [45–49].

**Table 3:** Statistical summary of MGWR local regression coefficients.

Variables	Mean coefficient	Std. deviation of coefficient	Minimum coefficient	Maximum coefficient	Median coefficient
***X***_***1***_	0.122	0.021	0.084	0.161	0.126
***X***_***2***_	−0.438	0.013	−0.472	−0.407	−0.437
***X***_***3***_	−0.001	0.055	−0.095	0.066	0.025
***X***_***4***_	−0.114	0.009	−0.391	−0.359	−0.364
***X***_***5***_	0.131	0.023	0.078	0.153	0.142
***X***_***6***_	0.127	0.01	0.096	0.139	0.131
***X***_***7***_	0.362	0.048	0.194	0.401	0.378
***X***_***8***_	1.323	0.014	1.284	1.355	1.325
***X***_***9***_	0.599	0.013	0.564	0.627	0.601
***X***_***10***_	−1.382	0.013	−1.419	−1.354	−1.38
***X***_***11***_	0.638	0.011	0.617	0.659	0.637

Note: *X*_*1*_ – number of bus stops; *X*_*2*_ – development intensity; *X*_*3*_ – land use mix; *X*_*4*_ – land use efficiency; *X*_*5*_ – distance to CBD; *X*_*6*_ – distance to bus stops; *X*_*7*_ – distance to hospitals; *X*_*8*_ – bus route density; *X*_*9*_ – residential density; *X*_*10*_ – building density; *X*_*11*_ – road density.

The MGWR coefficient regression results are visualized spatially in Fig 7. In the maps, the absolute value of the regression coefficient reflects the strength of influence each factor exerts on the equity of healthcare accessibility. A positive coefficient indicates a positive correlation between the dependent and explanatory variables—as the value of a given factor increases, the R-ENESA approaches 1, implying a narrowing difference between the two. Conversely, a negative coefficient suggests a negative correlation, where higher values of the explanatory variable correspond to greater disparities in accessibility between emergency and non-emergency care. The following patterns can be observed:

(1) Density Dimension: Urban development intensity tends to amplify disparities in healthcare accessibility, particularly in Zhenhai and Jiangbei, where high development intensity attracts dense population concentrations, generating greater demand for medical services. Similar regional disparities in healthcare facility distribution and accessibility have been reported in previous studies [6]. Building density also exerts a stronger influence on healthcare accessibility disparities in northern parts of Jiangbei and Zhenhai, with this effect gradually diminishing from the northern core urban areas toward the south. In regions of high building density, the clustering of production and residential activities leads to heightened medical demand, thereby exacerbating equity issues in access to care. Due to their greater flexibility and advantage in short-distance travel, EMS are less affected by building density, widening the gap between emergency and non-emergency care accessibility [45–49]. Additionally, residential density exerts a positive influence on accessibility disparities, with the most substantial impact observed in Zhenhai and a gradual weakening of this effect from north to south.
(2) Design Dimension: The number of bus stops plays a role in reducing disparities in healthcare accessibility. This effect is most pronounced in eastern Ningbo, gradually weakening toward the east. Similarly, road network density is significantly positively correlated with the reduction of accessibility disparities, particularly in the northern parts of Zhenhai and Beilun. This underscores road density’s crucial role as an essential component of urban infrastructure in promoting healthcare equity. The density of bus routes also has a positive impact on narrowing healthcare accessibility gaps, with the strength of this influence gradually decreasing from north to south. As the number of bus lines increases, travel time under non-emergency scenarios reduces, thereby narrowing the time gap between non-emergency and emergency healthcare access. The positive coefficients of all three indicators confirm that improvements in transport design make a positive contribution to healthcare equity under both emergency and non-emergency travel conditions [18,19].
(3) Destination and Distance Dimension: The distance to hospitals shows a consistently positive effect on accessibility disparities, with local regression coefficients ranging from 0.1935 to 0.4014, indicating that greater distances are associated with larger differences between emergency and non-emergency accessibility. This effect is more pronounced in the central-southern part of the urban core. In this area, the local regression coefficients range from 0.3866 to 0.4014, which are notably higher than those in other regions, indicating that the differences between emergency and non-emergency accessibility are relatively smaller. A possible explanation for this is that in the city center, the spatial distance to general hospitals is relatively short. As a result, travel time by private vehicles under emergency conditions is considerably lower than by public transport, leading to a greater disparity. In contrast, in southeastern peripheral mountainous areas, the greater spatial distance to hospitals and the constraints of road infrastructure prolong private vehicle travel times, narrowing the difference compared to public transportation. The distance to the CBD also shows a positive correlation with disparities in healthcare accessibility, with the influence gradually decreasing from northeast to southwest. This suggests that the closer residents are to the CBD, the more dominant private transportation becomes, and thus, the greater the disparity in healthcare accessibility between emergency and non-emergency scenarios. These patterns are primarily shaped by the city’s development strategy of “urban center first, suburban later,” which has concentrated more medical and commercial resources in the central urban areas [6].

**Fig 7:**
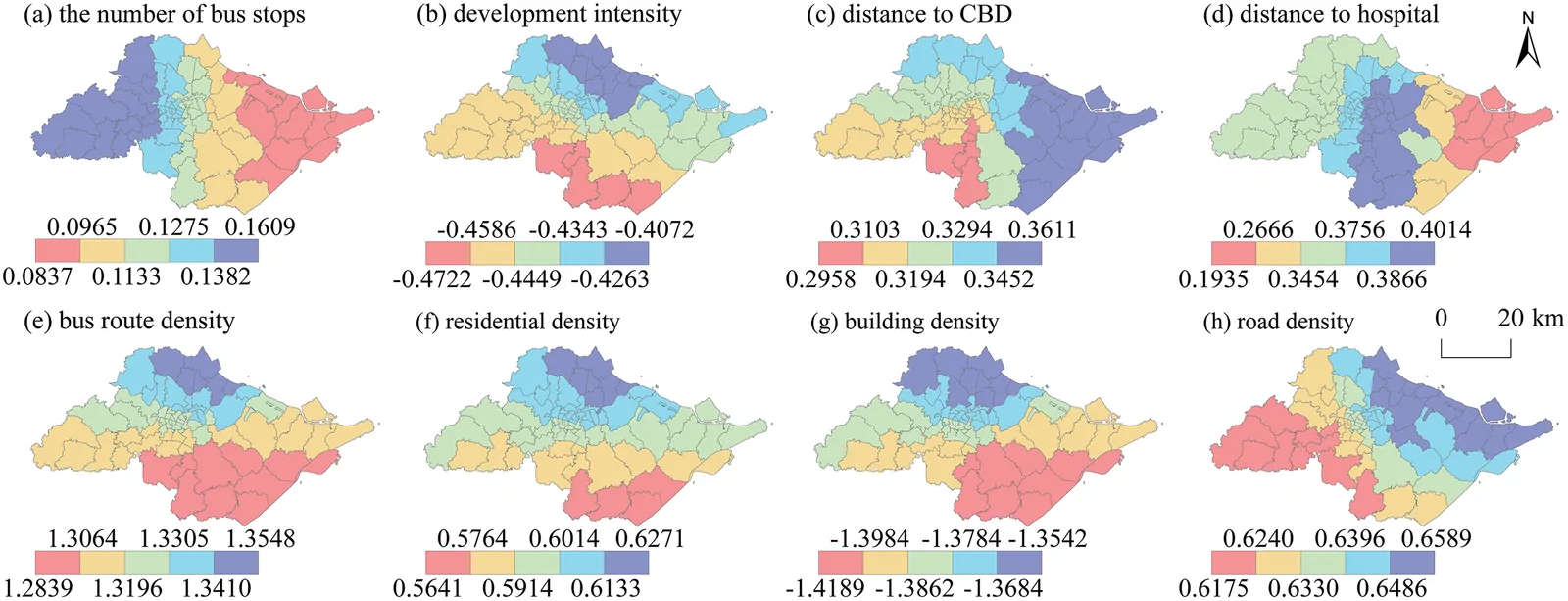
Spatial distribution of MGWR regression coefficients. Spatial statistics computed by the authors; administrative boundaries: Natural Earth (public domain); basemap: ©OpenStreetMap contributors (ODbL).

## Discussion and conclusion

### Strengths and limitations

This study analyzed the distribution characteristics and influencing factors of EMS in Ningbo from the perspective of spatiotemporal accessibility. The results differ somewhat from those of studies conducted in major domestic cities such as Shanghai [23] and Beijing [22,30], and Shenzhen [3] indicating that Ningbo’s EMS system still requires further development to gradually expand its coverage. At the same time, non-emergency medical accessibility based on public transportation remains underdeveloped, with 68.45% of areas requiring more than 60 minutes for the shortest trip. This severely hinders medical service equity. The spatial heterogeneity analysis reveals that the central urban area forms a low-low cluster, showing that EMS accessibility in the city center is better than in suburban and rural areas. This pattern aligns with findings from Shanghai’s inner districts [23] and Chongqing’s main urban area [16], confirming a positive correlation between urbanization levels and the concentration of medical resources. However, regional disparities in Ningbo are more pronounced, with some areas still facing shortages of high-quality EMS resources. Therefore, policymakers should pay particular attention to areas with poor EMS accessibility, increase fiscal investment in EMS in those regions, and enhance residents’ equitable access to EMS. Our findings can assist policymakers in improving the spatial accessibility of EMS in Ningbo. Furthermore, this research provides a reference for developing countries and regions to evaluate EMS transportation accessibility and optimize resource allocation. Although this paper was conducted in Ningbo, the approach of combining emergency with emergency medical travel time and measuring equity, as well as considering its spatial relevance, also applies to other studies.

This study has several limitations. First, it does not account for the temporal dynamics affecting medical accessibility. In particular, access to EMS may be significantly delayed during peak travel periods, a factor not explicitly addressed in this research [11,23]. Second, the shortest travel time approach provides a standardized measure of transportation accessibility but simplifies actual healthcare-seeking behavior. In particular, non-emergency patients may choose among multiple hospitals according to factors such as service capacity, medical specialty, hospital level, referral arrangements, and personal preference rather than travel time alone. Therefore, the accessibility estimates in this study primarily reflect potential transportation access under the specified travel scenarios. Third, the population data we used are based on the Seventh National Population Census, which may deviate from the current actual resident population of Ningbo. Furthermore, we did not analyze differences among population groups or socio-demographic characteristics within grid cells, such as the elderly, the disabled, or other vulnerable groups [5,7]. In this study, population was treated primarily as a spatial distribution variable rather than being classified by socio-economic attributes. As a result, potential variations related to population agglomeration, disadvantaged neighborhoods, or other demographic characteristics that may influence healthcare accessibility and alter the Lorenz curve were not explicitly considered. Finally, due to data constraints, this study is also limited to the central urban area of Ningbo and does not cover the entire municipal region. Future research could incorporate temporal variations in travel conditions, hospital service capacity and healthcare-seeking behavior, as well as accessibility differences among demographic groups, to provide a more comprehensive assessment of healthcare accessibility and its spatial equity.

### Main findings

This study used traffic data obtained from the API to investigate the accessibility of emergency and non-emergency medical services in the central urban area of Ningbo. The analysis incorporates the Lorenz curve and corresponding Gini coefficients to assess the spatial equity of EMS accessibility. In addition, global Moran’s I and LISA statistics were employed to measure the accessibility of general hospitals via public transportation and ambulances. The MGWR model was then used to analyze the spatial heterogeneity of influencing factors. The main findings are as follows:

First, there exist significant differences between emergency and non-emergency medical accessibility. We found that the shortest travel time for both emergency and non-emergency medical services increases outward from the city center. That is, medical accessibility decreases progressively from the urban core to peripheral areas, with the central zones demonstrating significantly better accessibility than outlying regions, particularly those in hilly fringe areas. This pattern is closely linked to the spatial distribution of general hospitals. The 15-minute service area for public transportation covers only 1.78% of the total area, while 68.45% of the region requires more than 60 minutes for the shortest travel time, highlighting an urgent need to improve public transit infrastructure. In contrast, ambulances cover 14.68% of the area within 15 minutes, with only 5.19% of the region exceeding 60 minutes of travel time, indicating substantially shorter modelled travel times under the emergency road-travel scenario than under the non-emergency public-transport scenario.

Second, there is a considerable disparity in the equity of emergency and non-emergency medical services. By establishing a functional relationship between population size and medical accessibility, we revealed that the Gini coefficients for both emergency and non-emergency modes exceed 0.4, indicating a relatively low level of equity. The Lorenz curves further reveal an uneven distribution of healthcare accessibility relative to the resident population. When the cumulative population reaches 60%, the corresponding cumulative accessibility shares are 23.77% under the emergency scenario and 25.02% under the non-emergency scenario. These patterns reflect a spatial mismatch between population distribution and access to medical facilities in the central urban area of Ningbo.

Third, medical accessibility exhibits significant spatial clustering characteristics. The global Moran’s I index at the township scale is greater than 0.8, and the LISA map shows evident clustering in high-value and low-value areas. The spatial correlation of shortest travel times is generally similar for both non-emergency and emergency modes. At the same time, the shortest travel time is negatively correlated with population density distribution. In the LISA map, the high-high type is primarily distributed across the hilly areas of the west and south, and is associated with factors such as complex terrain and long-distance general hospitals. Low-low types are more common in central built-up regions and are associated with higher economic levels and a greater degree of infrastructure development.

Fourth, the disparity in medical accessibility between emergency and non-emergency care, influenced by multiple factors and exhibiting significant spatial heterogeneity, is strongly explained by built environment elements. Factors such as the number of bus stops, distance to the CBD, distance to the nearest bus stop, distance to the nearest hospital, bus route density, residential density, and road density are positively associated with accessibility disparity—that is, increases in these variables are linked to greater differences in accessibility between emergency and non-emergency services. In contrast, building density, development intensity, land use efficiency, and land use mix have negative effects. Further spatial analysis reveals that the influence of bus route density, residential density, and building density decreases from north to south, while the number of bus stops has a particularly strong positive effect in the northwestern part of the city. These findings suggest that the optimization of transportation design plays a constructive role in improving equity in healthcare [10,11,19].

### Policy recommendations

This study reveals clear differences in the accessibility of emergency and non-emergency medical services in the central urban area of Ningbo. These differences should be interpreted in the context of the distinct service provision mechanisms of the two healthcare scenarios. Emergency accessibility is primarily influenced by the spatial organization of pre-hospital emergency resources, ambulance dispatch, road-network conditions, and the location of hospitals capable of receiving emergency patients, whereas non-emergency accessibility is more closely related to residents’ routine healthcare-seeking behavior, public transport conditions, and the spatial distribution of medical facilities [3,11,23,31]. Therefore, the observed differences between the two scenarios reflect not only differences in travel time but also the different ways in which emergency and routine healthcare services are organized and accessed.

For non-emergency healthcare, priority should be given to improving public transport connections between residential areas and general hospitals, particularly in peripheral areas with long travel times. The government should increase the coverage of bus stops, optimize bus route planning and transfer connections, and strengthen public transport links to major medical facilities [10,11,19]. Because non-emergency patients may select among different hospitals according to their healthcare needs rather than simply choosing the nearest facility [52], improving access to a broader network of medical facilities may help reduce spatial disparities in routine healthcare access. At the same time, the spatial coverage of medical facilities in underserved peripheral areas should be strengthened to reduce residents’ dependence on long-distance trips to the central urban area [6,23,24].

For emergency medical services, policy priorities should focus more directly on improving pre-hospital emergency response coverage and reducing transport delays. The spatial deployment of emergency stations and ambulances should be optimized according to population distribution and areas with persistently long emergency travel times, while coordination between emergency response facilities and receiving hospitals should also be strengthened [3,8,37]. In peripheral and mountainous areas, improving road connectivity and reducing critical transport bottlenecks can further enhance emergency response efficiency, as road-network structure and traffic conditions have been shown to play an important role in emergency medical accessibility [20–22]. Such differentiated interventions can better address the distinct accessibility constraints of emergency and non-emergency healthcare and contribute to a more equitable spatial distribution of healthcare access.
